# Predictors of sexual satisfaction among patients with chronic pain

**DOI:** 10.3389/fpain.2024.1375546

**Published:** 2024-04-04

**Authors:** Aex Barr, Kayla Moore, Lindsay G. Flegge, Emily Atsaphanthong, Krissa E. Kirby, Julia R. Craner

**Affiliations:** ^1^ Pain Rehabilitation Program, Mary Free Bed Rehabilitation Hospital, Grand Rapids, MI, United States; ^2^ College of Human Medicine, Michigan State University, Grand Rapids, MI, United States

**Keywords:** chronic pain, sexual satisfaction, biopsychosocial, antidepressant agents, perceived social support

## Abstract

**Objectives:**

Sexual satisfaction is an important aspect of quality of life. Chronic pain, depression and anxiety, and relational problems correspond with higher risk for sexual difficulties. Less is known about how risk factors for sexual dysfunction and other problems—such as medical conditions, pain severity, and medication side effects—affect the sexual satisfaction of people with chronic pain. Using a biopsychosocial framework, this study explored factors related to sexual satisfaction among patients presenting for evaluation of chronic pain.

**Methods:**

Researchers used a hierarchical multiple regression analysis to model potential predictors of sexual satisfaction. Variables analyzed were demographic features, medical history, average pain severity, depressed mood, anxiety, and perceived significant other support. Data collection involved administration of retrospective questionnaires and chart review. The sample included male and female participants (*N* = 134) presenting for evaluation at a multidisciplinary pain rehabilitation clinic.

**Results:**

Medical history (i.e., medical conditions, surgical history, and medications) and clinical self-report variables (i.e., pain severity, depressed mood, anxiety, and perceived significant other support) were associated with sexual satisfaction. In this sample, antidepressant use and higher pain severity were unique predictors of lower sexual satisfaction. Married marital status and higher levels of perceived significant other support were predictive of greater sexual satisfaction.

**Discussion:**

Findings highlight the importance of understanding the unique impact of biopsychosocial variables on the sexual satisfaction of patients presenting for evaluation at a multidisciplinary pain rehabilitation clinic. Further exploration of protective factors that account for sexual satisfaction among individuals with chronic pain may help inform screening, referrals, and treatment.

## Introduction

Sexual satisfaction is an important facet of overall quality of life in adults (1) and is strongly linked to relationship satisfaction (2, 3), physical health (1, 3, 4), mental health, and sexual functioning (3, 4). Sexual satisfaction depends on interactions among biological, psychological, and sociocultural factors (5). Chronic pain affects individuals across these domains (6). Chronic pain and sexual problems are also subjectively related. Of patients having completed a multidisciplinary pain rehabilitation program, 51.5% were shown to have 1 or more areas of sexual dysfunction, and 73% perceived these difficulties as “very much” or “somewhat” related to chronic pain (7). Sexual difficulties like sexual dysfunction and dissatisfaction are common and frequently co-occur with chronic pain. Research tends to focus on the *prevalence* of such sexual difficulties amid chronic pain. This paper contributes to a growing field of literature that considers biopsychosocial factors that predict sexual satisfaction among patients seeking treatment for chronic pain.

## Background

Individuals with chronic pain often have complex medical histories including multiple medical comorbidities [e.g., (8)]. Pain intensity is associated with increased sexual difficulties (9), and may be influenced by comorbid medical conditions. Chronic comorbid medical conditions may also have effects on sexual function due to symptoms, sequelae, and/or required medications associated with the disease. This can be the case with inflammatory bowel diseases like Crohn's disease (10), diabetes mellitus (11), epilepsy (12), cardiovascular and respiratory conditions (13, 14), and hepatic, urinary, and reproductive health conditions (15–17).

Addressing both biological and psychiatric risk factors for sexual difficulties among chronic pain patients can be complicated. On the biological level, the human sexual response entails coordination of numerous systems (e.g., cardiovascular, hormonal). Compromised functioning of these systems can affect libido, orgasm, and arousal-based responses and thus cause sexual dysfunction. Psychiatric problems such as depression and anxiety are prevalent and frequently comorbid with chronic pain. Stress, depression, and anxiety are known individual risk factors of sexual difficulties among people with chronic pain (18–20). Psychosocial and cultural influences may affect how individuals cope with medical (21) and psychiatric (22, 23) conditions, thereby affecting sexual function and perceptions of satisfaction.

Sexual dysfunction is a prominent side effect of certain first-line medications for common medical and psychiatric conditions as well as chronic pain. For instance, anticonvulsants (e.g., gabapentin) and antidepressants [e.g., tricyclics, specific serotonin, and serotonin-norepinephrine reuptake inhibitors (SSRIs, SNRIs)] are widely prescribed for chronic pain conditions (24). Antidepressants, anticonvulsants (25), antihypertensives (26), and long-term opiate use (27) have been linked to iatrogenic sexual dysfunction. Surgeries have also evidenced increased risk of sexual dysfunction (11, 28) and may alter or impede sexual engagement with partners.

Relationship factors are particularly relevant when investigating sexual satisfaction. Couples tend to report both reduced sexual and relational satisfaction associated with the onset of chronic pain (29). Conversely, relationship dissatisfaction may act as a long-term contributor to sexual difficulties amid chronic pain (18). Sexual difficulties have been found to predict relationship dissatisfaction among women with chronic pain and vice versa (22). Research has shown that preferences for social support correlate with pain-related disability (30). Perceived significant other support is particularly important for sexual wellbeing (22, 31) but has received less attention in treatment and the relevant literature.

Limitations of research on sexual satisfaction include both limited consideration of biological factors and exclusion of male participants (3). Few studies have examined specific predictors for sexual dissatisfaction in a clinically heterogenous sample of patients with non-malignant chronic pain [e.g., (32)]. However, many studies center a single condition that may be known to be associated with sexual difficulties, such as pelvic pain (33). Sexual satisfaction and chronic pain are complex, biopsychosocial experiences, and there is a deficit in clinical pain-related research that considers all these levels of experience simultaneously (6). This research is needed to elucidate the intricate relationships between sexual satisfaction and chronic pain, which could promote exploration of possible mechanisms of change and inform clinical interventions from a biopsychosocial framework.

The aim of this study was to examine predictors of sexual satisfaction among male and female individuals presenting for chronic pain evaluation. Based on prior research, the following variables were identified as potential risk factors: medical conditions, past surgeries, medication use, and endorsed anxiety and depression. Researchers hypothesized that lower levels of perceived support from significant others and higher levels of pain severity would be associated with higher levels of sexual dissatisfaction. These hypotheses were informed by an earlier study with preliminary data (34).

## Materials and methods

### Participants and recruitment

This study included retrospective data from both patient self-report questionnaires and chart review. Participants included 134 adults meeting the following inclusion criteria: (1) provided informed consent for study participation, (2) ≥18 years of age, (3) were presenting for a pain psychology evaluation at a multidisciplinary pain rehabilitation clinic, and (4) reported that they were sexually active within the past 30 days. Eligible patients included those seeking treatment at a 10- to 12- week chronic pain rehabilitation program, or seeking pain psychology services alone. Details of the multidisciplinary pain rehabilitation program have been previously described (35). Eligibility criteria are consistent with past studies on sexual function using similar samples (7, 36).

A total of 508 individuals were potentially eligible to participate in this study, 269 (53.0%) of whom consented to participate. Of those who consented, 247 completed measures related to sexual functioning, and 137 endorsed being sexually active. Three participants were excluded due to insufficient chart data to assess medical risk factors (*n *= 2) or ineligibility due to a presenting problem aside from pain (*n *= 1).

The Mary Free Bed (MFB) institutional review board (IRB) approved data collection encompassing use of patient self-report questionnaires and retrospective patient chart review. Participants were recruited using an electronic prompt following completion of electronic questionnaires given routinely at evaluation in a multidisciplinary pain rehabilitation clinic. Patients who completed the informed consent form were granted access to research questionnaires including information about sexual health and perceived social support. Patients who endorsed sexual activity alone or with partner(s) within the past 30 days were invited to answer additional questions about their sexual satisfaction during that period. Researchers conducted a patient chart review using electronic medical records of survey participants who were eligible for inclusion in analyses.

## Measures

Measures included self-reported pain severity, anxiety, depression, sexual functioning, and perceived social support and patient chart review. Researchers tested measure reliability using Cronbach's alpha coefficient. All measures had good internal consistency.

### Self-report questionnaires

#### Average pain severity

For the last 30 days, patients rated their average pain severity on a single-item, 11-point numeric scale ranging from 0 to 10. Numeric rating scales for pain have relatively good reliability (37), responsiveness, and ease of use (38). Higher scores suggest higher pain severity.

#### Anxiety

For the last 7 days, patients rated their anxiety symptoms using the Patient-Reported Outcomes Measurement Information System (PROMIS) Anxiety—Short Form 8a, an 8-item measure with item scales ranging from 1 (never) to 5 (always). Examples of items include “I felt anxious” and “My worries overwhelmed me.” This form has good validity and responsivity (39). For this and other PROMIS measures used in this study, raw total scores were converted to a *T*-distribution, with a mean (*M*) of 50 and standard deviation (*SD*) of 10. Higher *t*-scores are associated with higher anxiety symptom severity. Internal consistency (Cronbach's α) was high for the current sample (α = .95).

#### Depression

For the last 7 days, patients rated their depressive symptoms using the PROMIS Depression—Short Form 8a, an 8-item measure with item scales ranging from 1 (never) to 5 (always). Examples of items include “I felt depressed” and “I felt hopeless.” This form has strong validity and reliability (40) and moderate responsivity (41). Higher *t*-scores suggest higher depression symptom severity. Internal consistency was high (α = .95).

#### Sexual functioning

To assess domains of sexual wellbeing, the PROMIS Sexual Function and Satisfaction (SexFS) Version 2.0 (v2.0) was used. The PROMIS SexFS v.2.0 has strong validity and reliability (42). Examples of sexual activity assessed by this measure include sexual intercourse, masturbation, and oral sex. For the purposes of this study, satisfaction with one's sex life was used as the primary outcome variable (i.e., Global Satisfaction with Sex Life).

#### Female sexual function and satisfaction

Female participants completed the Brief Profile Sexual Function and Satisfaction (Female) 2.0. For the past 30 days, female patients rated their overall sexual function and satisfaction on a total 14 items. For the current study, only the Global Satisfaction with Sex Life subscale was used (2 items). Higher scores suggest higher satisfaction with sexual experiences. Internal consistency for this subscale was high in this study (α = .92).

#### Male sexual function and satisfaction

Male participants also received a sex-specific measure, the Brief Profile Sexual Function and Satisfaction (Male) 2.0, which pertains to sexual function and satisfaction over the past 30 days. This measure consists of 10 items. Only the 2-item Global Satisfaction with Sex Life subscale was used in this study. Internal consistency was high (α = .89).

#### Social support

The Multidimensional Scale of Perceived Social Support (MSPSS) (43) was used, which was designed to assess perceived social support. The MSPSS has good reliability, validity, and factor structure across multiple populations, including adults and psychiatric patients (44). Previous research has demonstrated a 3-factor structure consistent with its three distinctive subscales, indicative of perceived support from Family, Friends, and a Significant Other (45), respectively. The current study examined only the Significant Other subscale. Examples of items include “There is a special person in my life who cares about my feelings.”

### Chart review

The lead author developed a chart review method and trained and oversaw research assistants to ensure integrity of the chart review process. Information sources encompassed medical and pain psychology evaluations, scanned intake paperwork, and relevant chart documentation (e.g., referral forms, problem lists) as appropriate. Risk factors of interest were identified based on literature review. These factors included medications, past surgeries, and number and type of major medical co-morbidities. Demographic information regarding sex, relationship status (e.g., single, married), and living situation (e.g., living with spouse) were also obtained. Please refer to Table 1 for full list of chart review variables. Research assistants logged ambiguous items. To ensure information fidelity, the lead author conducted a secondary review of all charts marked as incomplete, inconsistent, or complex. Chart reviewers also noted conditions that they believed should be considered for statistical analyses, which the lead author reviewed and checked for relevancy. Subsequently, the lead author used a random number generator to select 18 out of 134 (13.43%) charts to assess interrater reliability. Each chart contained 23 categorical or numeric results, totaling 414 response items. Two items were found to have a different result by reviewer, suggesting 99.5% interrater agreement.

**Table 1:** Demographic and clinical characteristics.

Variable	*N* = 134
Age	
(*M* ± *SD*)	42.20 ± 13.23
Range (years)	18–76
Sex	
Female	94 (70.1%)
Male	40 (29.9%)
Marital status	
Never married	31 (23.1%)
Married	78 (58.2%)
Divorced	22 (16.4%)
Unknown/missing	3 (2.2%)
Relationship situation	
Single	22 (17.9%)
Cohabitating	101 (75.4%)
Living separately (not spouse)	3 (2.2%)
Separated from spouse	3 (2.2%)
Long distance/deployment	0 (0.0%)
Unknown/missing	3 (2.2%)
Race	
White/Caucasian	56 (41.8%)
Black/African American	10 (7.5%)
Other identification	1 (0.7%)
Unknown/missing	67 (50.0%)
Medical condition	
(*M* ± *SD*)	1.07 ± 1.17
Range (number of conditions)	0–6
Number of medical conditions	
0	53 (39.6%)
1	43 (32.1%)
2	21 (15.7%)
3	12 (9.0%)
4	4 (3.0%)
5	0 (0.0%)
6	1 (0.7%)
Medical condition by category	
Cardiovascular	18 (13.4%)
Urologic/reproductive	27 (20.1%)
Gastrointestinal	16 (11.9%)
Respiratory	45 (33.6%)
Diabetes	12 (9.0%)
Hepatic	5 (3.7%)
Neurological	4 (3.0%)
Surgery	23 (17.2%)
Antihypertensive meds use	45 (33.6%)
Antidepressant meds use	75 (56.0%)
Anticonvulsant meds use	53 (39.6%)
Opiate meds use	38 (28.4%)

## Data analytic strategy

The primary analysis for this study included a hierarchical multiple regression analysis to identify predictors of sexual satisfaction. Categorical variables were coded dichotomously prior to analyses. Sample size was determined appropriate to the analysis for testing individual predictors. Evaluation of statistical assumptions revealed no concerns with multicollinearity after assessing tolerance and variance inflation factors. Demographic features (i.e., age, sex, marital status) were entered in the first step of the analysis to account for variance attributed to these variables. Medical conditions (i.e., cardiac, urological/reproductive, gastrointestinal, respiratory, diabetic, hepatic, and neurological conditions and past surgeries) were entered in the second step; specifically, researchers examined the presence/absence of each medical condition category and total number of medical conditions. Finally, self-reported pain severity, depressed mood, anxiety, and perceived significant other support were entered in the third step. Significance was evaluated using a criterion of *p* < .05. Analyses were conducted using IBM SPSS version 28. A preliminary analysis was presented (46).

## Results

### Participant demographic characteristics

Participant age ranged from 18 to 76 years, with an average of 42.20 ± 13.23. Ninety-four individuals (70.1%) had female sex listed in their medical record, and the remaining 40 (29.9%) were listed as male. Regarding marital status, 78 (58.2%) participants identified as married, 31 (23.1%) as never married, and 22 (16.4%) as divorced. Most participants endorsed cohabitating with a significant other (*n *= 101, 75.4%) or single status (*n* = 22, 17.9%). Three (2.2%) participants identified as living separately from a partner (not a spouse), and another 3 (2.2%) indicated being separated from a spouse, respectively. No participants identified as having a long-distance relationship or a significant other deployed due to military service. Three (2.2%) responses to items about marital status and relationship situation were missing. Authors had racial identification information for half the sample, consisting of 67 patients (50%). Of this subsample, 56 (41.8%) were White/Caucasian, 10 (7.5%) were Black/African American, and 1 (0.7%) identified as another race. Table 1 summarizes demographic and clinical characteristics of this sample.

### Medical conditions and medication use

Of the full sample, 81 (60.4%) participants were found to have at least one major medical condition. The average number of major medical conditions was 1.07 ± 1.17, with a range of 0–6. In brief, 45 (33.6%) of participants had a respiratory condition, with asthma being the most common. Urologic/reproductive conditions were the second most common listed problem, affecting 27 (20.1%) participants. Additionally, 23 (17.2%) participants had had at least 1 surgery affecting urologic/reproductive health, with hysterectomy being predominant procedure documented. Less frequent conditions included major cardiovascular/cardiac conditions (*n = *18, 13.4%), diabetes (*n = *12, 9.0%), gastrointestinal conditions (*n = *16, 11.9%), hepatic conditions (*n = *5, 3.7%), and epilepsy or other listed major neurological issue (*n = *4, 3.0%). Supplemental Materials show specific medical conditions by frequency and medications included in these analyses.

Regarding antidepressant use, 62 (46.3%) of participants had one prescription listed, and another 9.7% had more than one antidepressant category documented (e.g., SSRI, SNRI). Anticonvulsants were the second most common prescribed drug class, with 53 (39.6%) participants having at least one prescription. Antihypertensive drugs were third most common, with 45 (33.6%) of participants having at least one prescription listed, and 17 (12.7%) of the total sample had multiple hypertensive drugs prescribed for concurrent use. Over one-in-four patients (*n* = 38, 28.4%) had a current prescription for opiates.

### Self-report measures

On the PROMIS Global Satisfaction with Sex Life subscale, the average *t-*score among female and male participants was 46.32 ± 9.59, with a range of 30.67–65.60. These data suggest that most patients indicated some sexual dissatisfaction, with 20.1% participants (*n* = 27) obtaining scores indicative of clinical levels of sexual dissatisfaction indicated by a score 1 SD below the mean (i.e., ≤40 *T*). Participants’ average depression rating was 60.95 ± 8.86, ranging from 37.1 ± 81.1, and average anxiety ratings were 62.58 ± 9.07, ranging from 37.10 to 83.00. These scores suggest participant endorsement of recent depression and anxiety symptom ranging from within normal limits (i.e., ≤55 *T*) up to severe (i.e., ≥70 *T*), with average ratings falling in the moderate range. Patients’ ratings of average pain ranged from 1 to 10, with an average of 6.32 ± 1.67. Average ratings on the MSPSS significant other subscale were 5.95 ± 1.44, with a range of 1–7. Correlations among measures are included in Table 2.

**Table 2:** Correlations among clinical self-report variables.

Measures	*M*	*SD*	1.	2.	3.	4.
1. PROMIS SexFS global satisfaction	46.32	9.59				
2. Average pain severity	6.32	1.67	−.26**			
3. PROMIS depression	60.95	8.86	−.33**	.35***		
4. PROMIS anxiety	62.58	9.07	−.34***	.32***	.76***	
5. MSPSS SO support	5.95	1.44	.33***	.10	−.24**	−.17*

*N *= 134.

* *p *≤ 0.05.

** *p *≤ 0.01.

*** *p *≤ 0.001.

### Predictors of sexual satisfaction

Results of the multiple hierarchical regression indicated that demographic variables (i.e., age, sex, marital status) accounted for 2.3% of the variance in the prediction of sexual satisfaction scores, *F*(3,130) = 2.05 *p* = .11. Medical history variables (i.e., medical conditions, surgical history, and medications) were entered on the next step. The addition of these variables added another 12.4% of accounted variance, *F*(15,118) = 1.60, *p* = .09. In the final step, clinical self-report variables were entered (i.e., pain severity, depressed mood, anxiety, significant other support), adding another 17.1% of explained variance, *F*(19,114) = 3.09, *p* < .001. Once all variables were entered, the final model accounted for 23.0% of the variance in sexual satisfaction scores. In the final model, the final variables were significant unique predictors of sexual satisfaction: marital status (*β* = .17, *p* = .04), antidepressant medication (*β* = −.19, *p* = .03), pain severity (*β* = −.28, *p* = .003), and perceived significant other support (*β* = .25, *p* = .01). Note that the direction of the relationships suggests that being married and having higher levels of significant other support was related to increased sexual satisfaction, whereas being on an antidepressant medication and having higher pain levels was associated with lower levels of sexual satisfaction. See Table 3.

**Table 3:** Hierarchical regression assessing predictors of sexual satisfaction.

Variable	*B*	*SE (B)*	*β*	Δ*R*^2^
Step 1: demographics				.05
Age	.005	06	.006	
Sex	−1.95	1.79	−.09	
Marital status	3.72	1.68	.19*	
Step 2: medical history				.12
Cardiovascular	4.36	2.54	.16	
Urologic/reproductive	.25	2.20	09	
Gastrointestinal	4.37	2.75	.16	
Respiratory	95	1.77	05	
Diabetes	.17	3.05	04	
Hepatic	.70	4.95	03	
Neurological	.65	5.45	08	
Surgery	1.80	2.46	.07	
Antihypertensive meds	.68	1.84	.03	
Antidepressant meds	3.96	1.76	.21*	
Anticonvulsant meds	.64	1.83	.03	
Opioid meds	.16	1.99	10	
Step 3: clinical variables				.17***
Pain severity	1.62	53	.28**	
Depressed mood	.04	14	.04	
Anxiety	.16	13	.15	
Significant other support	.63	57	24**	

Total *F*(19,114) for step 3 = 3.08***. Adjusted *R*^2^ = .23.

* *p* < .05.

** *p* < .01.

*** *p* < .001.

## Discussion

The aim of this study was to identify predictors of sexual satisfaction among sexually active patients presenting for a pain psychology evaluation in a multidisciplinary pain rehabilitation clinic setting. Findings demonstrated that antidepressant use and higher pain severity were unique predictors of lower sexual satisfaction. Being married and having greater levels of perceived significant other support were predictive of higher sexual satisfaction. These results demonstrate the significance of both biological and psychosocial risk factors for this aspect of sexual health among individuals presenting for evaluation of chronic pain. Consistent with prior literature, concurrent major medical conditions and elevated anxiety and depressive symptoms were common in this study population (10, 18, 19); however, these were not unique predictors of sexual satisfaction after controlling for other variables.

Antidepressant use stood out as an independent predictor among medical conditions, surgical history, and medications, jointly accounting for 12.1% of the variance in sexual satisfaction in this sample. This finding is remarkable given the high prevalence of antidepressant use in this sample (i.e., 46.3%), in contrast to 13.2% of US adults reporting use in the general community (47). This finding illustrates the importance of clinical assessment and education about the potential side effect profiles of antidepressants when prescribing them for pain control and/or psychiatric reasons in chronic pain populations. Notably, the prevalence of co-occurring medical and psychiatric conditions and polypharmacy may complicate approaches of offsetting sexual side effects (e.g., adjunctive medications) when working with patients with chronic pain.

As hypothesized, average pain intensity predicted sexual dissatisfaction. It is well established that pain severity is associated with risk for sexual difficulties in individuals with chronic pain. It is uncertain how effective pain treatment is for addressing multifactorial sexual dissatisfaction. Some research has demonstrated that reducing pain intensity is associated with improvements in sexual functioning (29). When pain is the primary problem affecting sexual activity, as may be the case in vulvodynia, pain reductions associated with medications have been shown to improve overall sexual function (48). A biopsychosocial approach involving a multidisciplinary pain rehabilitation team may best address concurrent chronic pain and sexual dissatisfaction in some cases.

Yet, best practices remain unclear. One study found that an interdisciplinary pain rehabilitation program (IPRP) resulted in self-reported improvement in sexual functioning for women with pelvic pain (49), while another suggested that an IPRP may be insufficient to improve sexual functioning in a sample of patients with heterogeneous pain conditions (7), despite prior evidence that IPRPs yield clinically significant reductions in pain and emotional distress [e.g., (50)]. Education and group interventions specific to sexual functioning have shown promising results (51). More research is needed to tailor sexual health interventions to individuals and relationships affected by chronic pain in multidisciplinary pain rehabilitation treatment settings.

This study shows an association between perceived significant other support and sexual satisfaction. Findings demonstrate that perceived significant other support may be associated with less emotional distress and more sexual satisfaction among individuals with chronic pain, regardless of pain severity. Prior studies demonstrated that less perceived support in a marriage is associated with more pain-related catastrophizing in individuals with long-term pain (31), and factors like empathic accuracy may be relevant to individuals’ evaluation of significant other support (52). How perceived significant other support relates to other relevant constructs, including relationship satisfaction [e.g., (32)], is not well understood. Overall, this study lends support to the view that relational factors and sexual satisfaction are critical to consider conjointly and adds to the literature in this area. Future research investigating significant other support, relationship satisfaction, and other partner dynamics is needed to elucidate the mechanisms that account for their association with sexual difficulties in populations with chronic pain.

In this study, marital status was a significant independent predictor of sexual satisfaction. Results are consistent with another study including a probability sample representative of the US adult population (*n *= 3,159) that found nonmarried women and men had significantly higher odds of sexual dysfunction when compared to married individuals (53). Most married participants in our sample identified as cohabitating with a spouse. This arrangement likely yields increased availability of a familiar sexual partner. Of note, the timing of this study corresponded with the ongoing COVID-19 pandemic, which may have affected partner availability, sexual behaviors, and sexual satisfaction for some [see (54, 55)]. Understanding reasons for sexual inactivity—whether due to personal preference, pain-related disability, etc.—would add valuable context to this study. It follows that research examining the prevalence of and reasons for sexual inactivity in a similar population may help interpret our findings.

More research using a strengths-based approach to sexual satisfaction among people with chronic pain would also be valuable. Prior research using similar samples found that participants presenting for this multidisciplinary pain rehabilitation clinic differed regarding primary presenting concern and program type (e.g., fibromyalgia, headache, complex regional pain syndrome, pain psychology only) (36). Most patients who completed a multidisciplinary pain rehabilitation program endorsed one or more areas of sexual dysfunction (7). Yet, 4 out of 5 patients in this sample did *not* report clinical levels of sexual dissatisfaction. This discrepancy suggests the existence of significant protective factors against sexual dissatisfaction amidst sexual dysfunction and various types of chronic pain. Our findings demonstrate that perceived significant other support may be one of those important protective factors.

Regarding sexual satisfaction, more research is needed to understand how patients seeking evaluation in a multidisciplinary pain rehabilitation clinic may differ from one another and the general population. Few studies have used the PROMIS SexFS to compare clinical and nonclinical populations [e.g., (56)]. Nonetheless, previous research has found that sexual satisfaction is linked to individual, partner, and relationship factors in the general community (57). This aligns with our findings that antidepressant use, marital status, and perceived significant other support are significant to sexual satisfaction in this sample. Comparing community samples with clinical pain populations may help clarify how pain intensity relates to these variables.

Age and sex were not independent predictors of sexual satisfaction in this sample. Yet, previous research has found gender, sex, and age relevant to sexual difficulties among individuals with chronic pain. In one study, lower sexual satisfaction was associated with higher pain severity, pain-related life interference, depression, and anxiety in female but not male participants (36). Another study found that women who reported higher depression, less relationship satisfaction, and older age reported lower sexual function, whereas age was the only significant correlate for sexual function in men (32). The current study included more biological variables than these investigations. Findings highlight the importance of accounting for biological, psychological, and social variables simultaneously while investigating demographic differences in sexual satisfaction among individuals with chronic pain.

Further research is needed to explore differences in sexual satisfaction among patients with diverse backgrounds seeking evaluation at a multidisciplinary pain rehabilitation clinic. Gender role expectations negatively affect pain perception and the quality of pain treatment for women (58). Race- and size-based oppression are correlated with higher pain intensity (59). How transgender and nonbinary patients with chronic pain define and achieve sexual satisfaction may differ from cisgender patients (60). Sexual orientation should be assessed in research given evidence of between-group differences in sexual satisfaction (61) and reported pain prevalence (62). Understanding the needs and experiences of marginalized and oppressed people can help address healthcare and pain disparities [e.g., (63–65)].

## Strengths and limitations

Strengths of this study include consideration of specific patient medical conditions, surgeries, and drug classes, which helps to inform understanding of the prevalence and complex relationships between these variables and sexual satisfaction in a sample of patients presenting for evaluation of chronic pain. This research also adds to the chronic pain literature considering individual psychological (i.e., depression, anxiety), pain-related (i.e., average pain severity) and relationship factors (i.e., marital status, relationship situation, significant other support) in the context of sexual satisfaction of individual chronic pain patients. These factors, most of which are subjective, accounted for more variance (i.e., 16.9%) than listed problems and medications in patients’ medical charts. Results highlight the importance of psychosocial variables regarding sexual satisfaction and suggest helpful directions for future research using a biopsychosocial approach to chronic pain and sexual satisfaction.

This study has several limitations. First, study generalizability is limited by characteristics of sample size and patient demographic characteristics (i.e., most were White/Caucasian, female, married, and all resided in the Midwestern United States). Unfortunately, race/ethnicity information was missing from 50% of the charts analyzed. Medications, specific pain conditions, and psychological conditions associated with increased odds of sexual dysfunction (e.g., antipsychotic drugs, serotonin antagonists and reuptake inhibitors) were not considered due to sample size. Relevant medication factors, including dosage and length of time of use, were also not considered for this reason. Because antihypertensive agents were a factor of interest in this study, hypertension itself was not analyzed as a potential risk factor due to the likely interaction of these variables; however, hypertension is known to contribute to sexual dysfunction (4).

These limitations may be addressed in future research to increase understanding of how these variables may relate to sexual satisfaction in patients with chronic pain. In addition, causality in these relationships cannot be established based on current findings, nor do these results rule-out the possibility of mediator or moderator variables. Future research examining the process by which marital status, perceived significant other support, antidepressant use, and pain intensity relate to sexual satisfaction would be a valuable addition to the literature in this area.

## Conclusions

Researchers conducted this study to investigate biopsychosocial variables accounting for sexual satisfaction among sexually active adult patients presenting for evaluation at a multidisciplinary pain rehabilitation clinic. About 1 in 5 participants obtained scores in the clinical range of sexual dissatisfaction. Relationship factors, antidepressant use, and pain intensity accounted for more variance in sexual satisfaction than noted comorbid medical issues. Findings highlight the importance of a biopsychosocial approach to research, clinical screening, and interventions to promote sexual satisfaction among patients with chronic pain presenting for evaluation. More research is needed to understand how best to address patient sexual dissatisfaction in a multidisciplinary pain rehabilitation clinic setting while accounting for the risk factors highlighted in this study. Research exploring how diversity factors and experiences of societal oppression relate to sexual satisfaction amidst chronic pain may be particularly valuable given known healthcare and pain disparities. Strengths-based research focused on factors such as perceived significant other support may also be helpful in gaining a comprehensive understanding of sexual satisfaction among individuals with chronic pain.

## Data Availability

The datasets presented in this article are not readily available because of the nature of the research. Where applicable, raw, anonymized data that support the findings of this study may be available from co-author Dr. JC, by reasonable request. Requests to access the datasets should be directed to julia.craner@maryfreebed.com.
