# Supplementary material for: Predictors of sexual satisfaction among patients with chronic pain
Source: Front Pain Res (Lausanne). 2024 Apr 4;5:1375546. doi: 10.3389/fpain.2024.1375546 (PMC11024270; doi:10.3389/fpain.2024.1375546)
Supplement: Supplementary file 1 [file Table1.docx]

| **Supplemental Materials** | | |
| --- | --- | --- |
| **Medical Condition Category** | ***N =* 134** | **Subcategories** |
| Diabetes  No  Yes | 122 (91.0%)  12 (9.0%) | None Specified |
| Number of Cardiovascular Conditions  0  1  2 | 116 (86.6%)  16 (11.9%)  2 (1.5%) | Congestive Heart Failure, Arrythmia, Heart Valve Problem, Angina, History of Myocardial Infarction, Cardiomyopathy, Thoracic Aortic Aneurysm |
| Number of Urologic/Reproductive Conditions  0  1  2 | 107 (79.9%)  24 (17.9%)  3 (2.2%) | Urologic/Stress Incontinence, Overactive Bladder, Kidney/Ureteral Stones, Interstitial Cystitis, Endometriosis, Prostatic Hyperplasia/ Prostatitis, History of Prostate Cancer, History of Bladder Cancer, Bladder Leakage, Neurogenic Bladder, Vaginal Vault Prolapse |
| Number of Gastrointestinal Conditions  0  1 | 118 (88.1%)  16 (11.9%) | Crohn’s Disease, Ulcerative Colitis, Inflammatory Bowel Disease, Gastroparesis |
| Number of Respiratory Conditions  0  1  2 | 89 (66.4%)  33 (24.6%)  12 (9.0%) | COPD, Asthma, Obstructive Sleep Apnea, Shortness of Breath with long COVID |
| Number of Hepatic Conditions  0  1 | 129 (96.3%)  5 (3.7%) | Hepatitis, Cirrhosis, Fatty liver disease, Hemochromatosis, History of Liver Cancer |
| Number of Neurological Conditions  0  1  2 | 130 (97.0%)  2 (1.5%)  2 (1.5%) | Epilepsy, Multiple Sclerosis, Hypoglossal Nerve Problem |
| Number of Surgeries  0  1  2 | 111 (82.8%)  21 (15.7%)  2 (1.5%) | Splenectomy, Low Anterior/Abdomino-perineal Resection, Cystectomy, Prostatectomy, Hysterectomy, Oophorectomy |
| Antihypertensive Drug Use  0  1 Class  2 Classes  3 Classes  4 Classes | 89 (66.4%)  28 (20.9%)  11 (8.2%)  5 (3.7%)  1 (0.7%) | Alpha Blockers/Inhibitors: Doxazosin, Finasteride, Prazosin, Phenoxybenzamine, Silodosin, Tamsulosin, Terazosin hydrochloride, Uroxatral  Angiotensin II blockers: Azilsartan, Candesartan, Eprosartan Mesylate, Irbesartan, Losartan, Olmesartan, Telmisartan, Valsartan  Calcium channel blockers: Amlodipine, Clevidipine Butyrate, Diltiazem, Felodipine, Isradipine, Levamlodipine, Nicardipine, Nifedipine, Nimodipine, Nisoldipine, Verapamil  Thiazide(-like) diuretics: Bendroflumethiazide, Chlorothiazide, Chlorthalidone, Hydrochlorothiazide, Indapamide, Methyclothiazide, Metolazone  Loop diuretics: Bumetanide, Ethacrynic acid, Furosemide, Torsemide  Potassium-sparing diuretics: Amiloride, Eplerenone, Spironolactone, Triamterene  Beta Blockers: Acebutolol, Atenolol, Betaxolol, Bisoprolol, Carvedilol, Carteolol, Esmolol, Labetalol, Levobunolol, Metipranolol, Nadolol, Nebivolol, Metoprolol, Penbutolol, Pindolol, Propranolol, Sotalol, Timolol  ACE Inhibitors: Benazepril, Captopril, Enalapril, Fosinopril, Lisinopril, Moexipril, Perindopril, Quinapril, Ramipril, Trandolapril  Amlodipine-Benazepril Combination* |
| Antidepressant Drug Use  0  1 Class  2 Classes  3 Classes | 59 (44.0%)  62 (46.3%)  11 (8.2%)  2 (1.5%) | MAOI: Isocarboxazid, Phenelzine, Selegiline, Tranylcypromine  SSRI: Citalopram, Escitalopram, Fluoxetine, Fluvoxamine, Paroxetine, Sertraline, Vilazodone  SNRI: Desvenlafaxine, Duloxetine, Levomilnacipran, Milnacipran, Venlafaxine  Tricyclics: Amitriptyline, Amoxapine, Clomipramine, Desipramine, Doxepin, Imipramine, Nortriptyline, Protriptyline, Trimipramine  Other: Vortioxetine |
| Anticonvulsant Drug Use  No  Yes | 81 (60.4%)  53 (39.6%) | Carbamazepine, Phenytoin, Sodium Valproate, Clobazam, Clonazepam, Gabapentin, Pregabalin, Topiramate, Tiagabine |
| Opiate Drug Use  No  Yes | 96 (71.6%)  38 (28.4%) | Alfentanil, Buprenorphine, Butorphanol, Codeine, Fentanyl, Hydrocodone, Hydromorphone, Levorphanol, Meperidine, Methadone, Morphine, Nalbuphine, Oliceridine, Opium, Oxycodone, Oxymorphone, Pentazocine, Propoxyphene, Remifentanil, Sufentanil, Tapentadol, Tramadol |

***counted as 2 classes of antihypertensives
